# Bupropion-Induced Diplopia in an Iranian Patient

**Published:** 2011

**Authors:** Mohammad Reza Fayyazi Bordbar, Morteza Jafarzadeh

**Affiliations:** 1 Associated professor of Psychiatry, Psychiatry & Behavioral Sciences Research Center, Psychiatry Department, Mashhad University of Medical Sciences, Mashhad, Iran.; 2 Resident of Psychiatry, Psychiatry & Behavioral Sciences Research Center, Psychiatry Department, Mashhad University of Medical Sciences, Mashhad, Iran.

**Keywords:** Bupropion, Depression, Diplopia

## Abstract

We present a case report of bupropion-induced diplopia in a 26-year-old woman who suffered from atypical depression. After four weeks of taking bupropion (225 mg per day) she complained of headaches, blurred vision, and diplopia. No neurological and ophthalmologic abnormal signs were found. Her brain magnetic resonance imaging (MRI) and magnetic resonance venography (MRV) were normal as well. After tapering off and discontinuing bupropion, her diplopia resolved within a week.

## Introduction

Bupropion was invented in 1969 by Nariman Mehta in the hopes of developing a superior antidepressant with abilities to treat various psychiatric disorders (1). Its use is extensive and its safety is well established. Bupropion is a unicyclic aminoketone that resembles amphetamine and the anorectic diethylpropion in its molecular structures (2,3). Bupropion is a dopamine and norepinephrine reuptake inhibiter and is a unique antidepressant in the available armamentarium of drugs, with a highly favorable profile of adverse effects (4). Of particular note among antidepressants, it is associated with little inhibition of sexual function. It also carries a higher likelihood of weight loss than weight gain. It possesses some dopaminergic effects and may serve as a mild psychostimulant as well as an antidepressant (5).

We found no reports on bupropion and diplopia in Iran. Information on bupropions side effects is available from controlled and uncontrolled studies as well as post marketing surveillance reports. The most common side effects reported with bupropion are agitation, anxiety, and insomnia which most often occur during the initial stages of bupropion therapy. Other relatively common side-effects reported with bupropion include fever, dry mouth, headache or migraine, dizziness, nausea and vomiting, constipation, urinary frequency, tremor, sweating and skin rashes. Hypersensivity reactions, tachycardia, chest pain and hypertension (sometimes sever), vasodilatation, postural hypertension, palpitation, syncope, psychotic episodes, confusion, nightmare, impaired memory, dysgeusia, anorexia with weight loss, paraesthesia, tinnitus and visual disturbances have also been reported (3,6).

To date, the ocular side effects reported with bupropion include abnormal accommodation, blurred vision, glaucoma, increased intraocular pressure and dilated pupils. Rare reports of diplopia have been noted with bupropion therapy as well (6). These reports are all related to slow-released (SR) formula and high doses of bupropion. We present the case of a patient with atypical depression who developed diplopia while treated with bupropion. Reviewing the literature, we found no report concerning occurrence of diplopia as a result of using simple form and low or moderate doses of bupropion. What is reported here, thus, is believed to be the first case of diplopia due to administration of moderate dosage of bupropion.

## Case Report

The patient was a 26-year-old woman who presented to our psychiatry clinic in winter 2010. Her past medical history consisted of adjustment disorder four years earlier accompanied by depressed mood for a period of 6 months. Then, she experienced a period of major depressive disorder with atypical features. In this period she suffered from deep sadness, isolation, hypersomnia, declining concentration, craving for sweets, thought rumination with feelings of worthlessness and self-accusation as well as dysfunction at workplace. However, she was not anxious. Flouxetin was administered for her with a dose of 10 mg/day which then its dosage was doubled in a week. After 3 months due to low compliance, no significant recovery was observed regarding her symptoms. She was then received cognitive-behavioral-therapy. Although the symptoms were not fully regressed, the patient could show signs of improvement in different psychological areas.

During the past several months before her last presentation, she experienced worsening of her depressed mood. Her depressed mood worsened and she gained weight and craved for sweets a lot. Sleeping a lot, decrease in concentration and sexual drive and isolation were among other her symptoms. Flouxetin was tapered off and the patient started taking bupropion (Abidi Brand) (75 mg/day) which was increased to 150 mg/day in a week and to 225 mg/day in two weeks. Two weeks after the last dose increase, the patient complained of headaches, blurred vision, and diplopia. As diplopia was not among the reported side effects of bupropion, neurological consultation was carried out to know about the possible underlying causes. Neurological consultation did not reveal any neurologic abnormality. Her brain magnetic resonance imaging (MRI) was normal as well. Ophthalmologic consultation was also done which was normal and her true diplopia was approved. Magnetic resonance venograghy (MRV) was also normal. Due to not finding a reason for her diplopia, bupropion was tapered off and discontinued in 3 days. Following discontinuation of bupropion, the patient’s diplopia resolved within a week. Neurological and ophthalmologic examinations were done after 3 weeks and no abnormal finding was reported.

## Discussion

The authors reported a rare side effect of bupropion, diplopia, in a young female who received this medication for approximately one month. Based on neurologic and ophthalmologic examinations and because there was no medical reason to observe diplopia which resolved after discontinuation of bupropion, a relation between this side effect and bupropion is likely. Though such a side effect has not been reported in the Iranian literature to date, we would assume that using bupropion with a dosage of 225 mg daily has caused diplopia in the young patient.

Some authors have reported diplopia with taking high doses (300 to 400 mg) of SR bupropion. Yet these results are not higher than the reports in case of using placebo. In a study that aimed at comparing the side effects of this medication with doses of 300 mg a day (n = 376), 400 mg a day (n = 114) and placebo (n = 385), occurrence of diplopia was reported as 3% in the first group and 2% in the last group (7).

This report provides the first documented case of diplopia after using bupropion in a depressive patient. Though visual problems after taking bupropion are quite rare, tracing such a problem in this case following a fairly low daily dose highlights the importance of the issue.

## Authors' Contributions

Both authors have been involved in the acquisition of clinical data and in the reviewing the scientific literature. MRFB wrote the manuscript. Both authors read and approved the final manuscript.

## Notes

**Declaration of interest**: None.
